# Recent Progress in Distributed Fiber Optic Sensors

**DOI:** 10.3390/s120708601

**Published:** 2012-06-26

**Authors:** Xiaoyi Bao, Liang Chen

**Affiliations:** Physics Department, University of Ottawa, Ottawa, ON K1N6N5, Canada; E-Mail: lchen@uottawa.ca

**Keywords:** fiber optic sensors, brillouin scattering, Rayleigh scattering, Raman scattering, distributed sensors, birefringence, temperature, strain, vibration, optical time domain reflectrometer (OTDR), optical frequency domain reflectrometer (OFDR)

## Abstract

Rayleigh, Brillouin and Raman scatterings in fibers result from the interaction of photons with local material characteristic features like density, temperature and strain. For example an acoustic/mechanical wave generates a dynamic density variation; such a variation may be affected by local temperature, strain, vibration and birefringence. By detecting changes in the amplitude, frequency and phase of light scattered along a fiber, one can realize a distributed fiber sensor for measuring localized temperature, strain, vibration and birefringence over lengths ranging from meters to one hundred kilometers. Such a measurement can be made in the time domain or frequency domain to resolve location information. With coherent detection of the scattered light one can observe changes in birefringence and beat length for fibers and devices. The progress on state of the art technology for sensing performance, in terms of spatial resolution and limitations on sensing length is reviewed. These distributed sensors can be used for disaster prevention in the civil structural monitoring of pipelines, bridges, dams and railroads. A sensor with centimeter spatial resolution and high precision measurement of temperature, strain, vibration and birefringence can find applications in aerospace smart structures, material processing, and the characterization of optical materials and devices.

## 1. Motivation for Distributed Fiber Sensors

When one drives over a bridge, or through a tunnel, or stays inside a large building or takes an airplane, or uses the power from water dams or power generators, we want to be confident about the health of those structures. They can be inspected from the outside, but internal stresses and strains affecting their frameworks remain all but impossible to measure in a practical efficient way. Detecting cracks in concrete before they become visible could help prevent structural collapse of buildings and other civil works. Therefore, diagnosing the health of structures has been an ultimate driving force for the development of distributed fiber sensors.

A truly distributed optical sensor is expected to reveal temperature, strain and vibration information from any point along an optical fiber through light scattering. The challenge has been to find a mechanism that would allow the key structural parameters to be determined at any point along an optical fiber with an appropriate sensitivity and spatial resolution, and yet within acceptable time limits for vibration or dynamic strain detection. Fortunately, due to the great efforts of researchers from the fiber sensor community over the last 20 years, the performances of distributed strain or temperature sensors are adequate for many applications that require large areas of coverage with high location accuracy [1], especially for distributed sensors based on Brillouin scattering, the strain resolution is a few micro-meters over one meter (micro-strain) and the temperature resolution can be less than 1 °C [2]. The standard communication fibers can be imbedded in structures such as bridges, buildings, dams, power generators, airplanes and other civil works, to report their internal status.

Fiber optic systems will become one of the core technologies to reveal local information of various structures as a function of the monitoring probe, and they can be combined with instrumentation technology to assist us in making decisions on the safety of each structure. The link from a local probe to the decision maker would be provided by Internet through telecommunications. This will reduce the civil structure danger and their fatal impacts on our daily lives.

Distributed sensors can also find applications in fiber optic communication field, where spatially resolved measurements of polarization properties of fiber optic link on the birefringence, polarization-mode dispersion (PMD) could be conducted with polarization sensitive reflectometric techniques. The importance of such a monitoring is to provide guideline on design and manufacturing low PMD fibers to enable high speed communication systems.

This paper reviews various distributed sensors that have been developed based on Rayleigh, Brillouin and Raman scattering, and their working principles, as well as some of the applications. The paper is arranged as follows: the introduction and outline is contained in Section 1; followed by the definition and system limitation in Section 2, Section 3 then covers the theory and working principle of spontaneous Rayleigh, Brillouin and Raman scattering, as well as their mechanisms for measuring strain and temperature; the use of the Stokes and anti-Stokes ratio in Raman scattering for distributed temperature sensing is explained in Section 4; the Rayleigh scattering based OTDR (optical time-domain reflectometry) [3] and OFDR (optical frequency-domain reflectometry) and system performance are illustrated in Sections 5 and 6 along with discussion of vibration, acoustic wave and birefringence measurement using coherent detection of phase OTDR and polarization OTDR (POTDR), as well as device characterization using the OFDR technique; Section 7 discusses Brillouin scattering based distributed sensors, which have been intensely studied for last twenty years along with their various applications. In this section, we shall review major milestones associated with Brillouin Optical Time Domain Reflectometry (BOTDR) and Brillouin Optical Time Domain Analysis (BOTDA); followed by the use of combined Brillouin gain and loss to form parametric gain to make distributed sensors, the phase matching conditions for the gain and loss process, and their different Brillouin frequencies due to the chromatic dispersion (CD) and PMD, and the potential applications to measure CD and PMD using the off-resonance Brillouin spectrum. Recent development of the Brillouin grating, differential Brillouin gain and Brillouin “echo” have been discussed in this section as well, along with summarizing Brillouin scattering based distributed sensors operating in the frequency domain. In Section 8 we will review the progress of a new type of Brillouin grating based sensor. Sections 9 and 10 provide a summary of distributed vibration measurement and a performance chart of distributed sensors, discussing limitations on sensing length, as well as spatial, temperature and strain resolution, and they provide a performance chart of different sensing systems as a function of different performance parameters. In Section 11 we discuss the applications of distributed sensors for monitoring the structural condition, and Sections 12 and 13 present the overall summary and conclusions of this paper.

## 2. Definition of Distributed Sensors

This section explains the concept of distributed sensors based on Rayleigh, Brillouin and Raman scattering, and how the time domain and frequency domain signal can be used to get location information in the fiber to provide distributed information on temperature, strain and vibration. It also gives a brief discussion on the limitations of distributed sensors.

When an electromagnetic wave is launched into an optical fiber, the light will be redistributed by various mechanisms in the form of Rayleigh, Brillouin or Raman scattering. If the local temperature, strain, vibration and acoustic wave changes are relayed to (mostly via direct contact with some types of specialty glue) the optical fibers, the scattered signal in the fiber will be modulated by these physical parameters, and by measuring the changes of modulated signal, one can realize fiber sensors. If the input light is a pulsed signal with a pulse width of *τ*, then the location of the modulated signal along the optical fiber by temperature or stress change can be measured by the time delay of the speed of light *c*, the location accuracy is called spatial resolution:

(1) $$\Delta z=\frac{\tau C}{2n_{\text{eff}}}$$

where *n_eff_* is the effective refractive index of the fiber, which is associated with group index. The factor of 2 is attributed to the travel time of incoming pumped and scattered light. The pulse width defined spatial resolution is commonly used in Rayleigh scattering based OTDR test sets. Rayleigh scattering is an elastic scattering process with no frequency shift and the pulse spectral width is in the range of MHz, hence the group index and phase index variation within the spectral width can be neglected. However for the different frequency shifts associated with Brillouin (GHz) and Raman (THz) scattering, the variations of phase and group indexes are not negligible, especially for fibers with high chromatic dispersion (CD) and PMD with separated Stokes components in fast and slow axis, particularly over long fiber lengths (>10 km).

In the field of distributed fiber optic sensors, another definition is commonly used for spatial resolution, *i.e.*, 10–90% rise time of a transition of measurand [4]. This measurement should be carried out at the end of the sensing length where the signal to noise ratio is the worst, so that the claimed spatial resolution is effective for the entire sensing length. Ideally the stress or temperature section equivalent to the spatial resolution at the end of the sensing length should be demonstrated to show the capability of the distributed sensors. The sensing coverage length is determined by the fiber length, the so called sensing length *L*.

In the specification of commercial systems, read-out resolution is often given; this refers to the spatial distance between two neighboring points in the time domain, which is determined by the sampling rate of the digitizer. For instance, a 20 G sample/s digitizer gives the time separation of 50 ps, the equivalent spatial distance in optical fiber is 5 mm. In this case the read-out resolution is 5 mm. However the definition of 10–90% signal change at a transition point of temperature or strain variation includes the convolution of the bandwidths of photo detector, electrical amplifier and digitizer, as well as rise-time of the pulse generator associated bandwidth. Obviously the spatial resolution gives the capability of a distributed sensor to discretize a gradient and identify localized changes in a strain or temperature distribution.

In general, a distributed sensor can replace many point sensors. As a result it is the most cost effective, and weight and space efficient sensor system available, as it only requires one fiber capable of sending and receiving the signal from *the same fiber* and only one monitor is adequate to display the local changes in temperature, stress, vibration and acoustic waves. This considerable light weight advantage makes distributed sensors based on light scattering in optical fiber the most powerful monitoring option even in comparison to point fiber sensors, especially for structural monitoring when the distribution change is critical for a civil or aerospace structure.

In addition to time domain distributed sensors which use optical pulse to determine the spatial resolution as 10–90% changing signal in time domain as described above, there are three types of OTDR sensors based on Rayleigh [3], Brillouin [5] and Raman [6] scattering.

Distributed sensor can also be realized in the frequency domain, so called OFDR based on Rayleigh scattering [7] and Raman scattering of the power ratio of Stokes and anti-Stokes [8]. The Raman technology is based on frequency modulation of an electrical optical modulator's baseband signal, the detection of Stokes and anti-Stokes ratio then gives a temperature relation, due to the small tuning range of the modulator; this technology yields a spatial resolution of 1 m [9] over 1 km length using direct detection without getting phase information.

Rayleigh based OFDR uses a tunable laser to scan a frequency range of Δ*F* and through Fourier transformation produces a spatial resolution of:

(2) $$\Delta z=\frac{c}{2n_{\text{eff}}\Delta F}$$

By increasing the frequency scanning range, the spatial resolution can be reduced to a millimeter or less. The sensing length is limited by the coherence length of the laser source and the state of polarization change along the fiber, which is tens of meters. Early development of OFDR was focused on locating high attenuation points [7] because OFDR systems can give high spatial resolution (on the order of 1 mm). The sensing length is limited by the coherence length of the tunable laser (<100 m) [10]. Recently this was further explored by adding inverse Fourier transformation to recover frequency dependent temperature or strain changes in one small fiber segment [11], which makes OFDR as a sensor based on Rayleigh scattering. Additionally, OFDRs are generally based on an interferometric technique, which means it can be used for relative refractive index measurement in a distributed form. OFDR sensors tend to be sensitive to bending loss, so for civil structural monitoring, fibers must be protected from sharp bends. Although OFDR often uses single mode fiber, it can also be implemented with multi-mode fiber for differential mode delay measurement [10], and for mode coupling monitoring in tapered fiber [12].

Distributed fiber optic sensors can be used to monitor the variation of civil and aerospace structural conditions with spatial resolutions ranging from one millimeter to several meters, and the sensing length varies from meters to over 100 km [2]. Such a wide range of sensing coverage and spatial resolution would enable varying applications from optical device characterization, to monitoring of large civil and aerospace structural conditions.

## 3. Spontaneous and Stimulated Scattering in Optical Fibers

This section will be focused on the mathematical derivation of Rayleigh, Brillouin and Raman scattering and the relation of the scattered light to temperature, strain and stress for sensing purposes. The concepts of birefringence and nonlinear scattering in the context of distributed sensing will also be discussed.

When a light wave propagates in a medium, it interacts with the constituent atoms and molecules, and if its wavelength is far from a medium resonance, the electric field induces a time dependent polarization dipole. The induced dipole generates a secondary electromagnetic wave, and this is so called light scattering. Because the distances between scattering centers (particles) are smaller than the wavelength of light in the optical fibers, the secondary lightwaves are coherent for Rayleigh scattering. Hence, the resulting intensity is the addition of the scattered fields.

When the medium is perfectly homogeneous, the phase relationship of the emitted waves only allows the forward scattered beam. The optical fiber is an inhomogeneous medium, scattering arises from microscopic or macroscopic variations in density, composition or structure of a material through which light is passing. The random ordering of the molecules and the presence of dopants cause localized variations in density (and therefore refractive index). These give rise to Rayleigh scattering which causes attenuation of the forward-propagating signal (and creation of a backward-propagating wave) that is proportional to $\frac{1}{\lambda^{4}}$.

**Figure 1.**
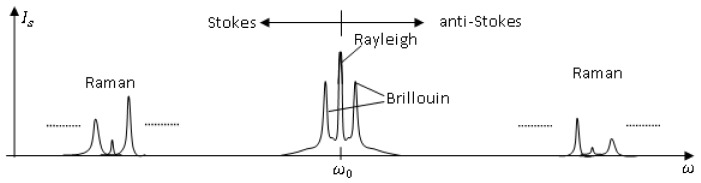
Typical spontaneous scattering spectrum from solid state matter.

Rayleigh scattering is a linear scattering process in that the scattered power is simply proportional to the incident power. Also, no energy is transferred to the glass in Rayleigh scattering, therefore there is no change in frequency of the scattered light comparing with that of the incident light, so called *elastic scattering*. It is attributed to non-propagating density fluctuations [13]. In Figure 1, the two lines appearing on both sides of the Rayleigh peak are the Brillouin lines. They are contributed by the scattering of sound waves moving in opposite directions. The left peak with a downshifted frequency is called the Stokes peak, while the right one with an up-shifted frequency is called the anti-Stokes line. Raman lines are contributed by the interaction of the lightwave with molecular vibrations in the medium. Both Brillouin and Raman scattering are *inelastic* scattering because they are associated with some frequency shifts. The last mechanism that can be observed is the Rayleigh wing scattering attributed to fluctuations in the orientation of anisotropic molecules. Raman spectra usually contain many sharp bands with separations between bands corresponding to the electronic vibrations and each bandwidth results from molecular rotation or reorientation excitations.

As long as the input light is scattered without strongly altering the property of the medium, we will say that the scattering is spontaneous, which includes Rayleigh, Brillouin and Raman scattering. When the light intensity increases to a level such that the optical property of the medium is modified, and the scattered light is proportional to the power of the input light, then this regime becomes stimulated. In other words, the evolution from spontaneous to stimulated scattering corresponds to a transition of the medium behavior from a linear to a non-linear regime.

### 3.1. Rayleigh Scattering

**Figure 2.**
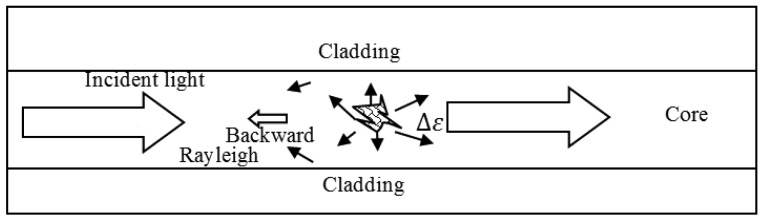
Schematic diagram for the spontaneous Rayleigh scattering process.

On the microscopic level the molecules making up any ordinary matters are immersed in a violent internal electromagnetic (EM) environment in spite of the macroscopic charge neutrality as is true for most macroscopic materials. Those violent EM environments are constantly causing the molecule to readjust its electron clouds. By changing its own electron cloud configuration this molecule is contributing to the changing environment for other neighboring molecules in a perpetual cycle. Therefore on a relatively small spatial scale (order of tens of molecular sizes) one would observe fluctuations in terms of local charge density, local temperature or even strain values. Without incident light such short range fluctuations would not produce measurable macroscopic effects at a far distance, as they are mutually incoherent and thus cancelled out. In this case the macroscopic EM fields inside any material are zero. However, with external light incident on a material, this EM field ***E*** will reorient the originally incoherent random fluctuating molecular clouds such that there is a tendency to respond collectively the same way on a small spatial scale covering a small fraction of the wavelength of the EM field. Such a collective tendency to respond to an EM field would result in macroscopic polarization that is proportional to the external electric field ***E, P*** = *∊*_0_*χ**E***. The parameter *χ* is a material status dependent quantity characterizing the collective response; the value *χ* possesses a randomly fluctuating portion Δ*ε* (*t, z*). This fluctuating dielectric parameter Δ*ε* gives a fluctuating polarization-induced light emission in all directions as illustrated in the Figure 2. Some of the scattered Rayleigh light is re-captured by the waveguide and sent in the backward direction. This backward propagating Rayleigh scattered light has a time delay that can be used for distributed sensing. The Rayleigh scattering can be treated as a single scattering process. Hence an OTDR trace can be used to locate fiber components in a network, and as vibration sensors based on phase OTDR described in Section 5, and temperature and strain sensing based on OFDR as described in Section 6.

Assuming simple linearity of the polarization for a non-magnetic material like the fiber, we write the electric displacement field vector [14]:

(3) $$\text{D}={∊}_{0}(1+\chi )\text{E}=(\varepsilon +\Delta ∊)\text{E}$$

(Here *ε* is a constant while Δ*∊* describes the locally fluctuating physical mechanism, *i.e.*, spontaneous scattering. For the case of Rayleigh scattering one assumes that Δ*∊* varies spatially. Using the Maxwell equation we can get following electric field **E**:

(4) $$\mu_{0}\varepsilon \frac{\partial^{2}\text{E}}{\partial t^{2}}-\nabla^{2}\text{E}-\nabla [\text{E}\cdot \nabla \ln(\varepsilon +\Delta ∊)]+\mu_{0}\frac{\partial^{2}(\Delta ∊\text{E})}{\partial t^{2}}=0$$

The first two terms in Equation (4) describes the ordinary coherent propagation process, while the third and the fourth terms describe the random spontaneous scattering terms caused by the fluctuation Δ*∊* that is both time and spatially dependent. To further simplify the physics we can assume Δ*∊* to be time independent (*i.e.*, consider the Rayleigh scattering only), and then one can replace the partial time derivative (assuming a time variation of the complex form *e^−iωt^* for the incident **E** field), $\frac{\partial }{\partial t}\to -i\omega$. Hence the Maxwell equation can be further simplified:

(5) $$\nabla^{2}\text{E}+\nabla [\text{E}\cdot \nabla \ln(\varepsilon +\Delta ∊)]+\mu_{0}\varepsilon \omega^{2}\left(1+\frac{\Delta ∊}{\varepsilon }\right)\text{E}=0$$

For the case of optical fiber, we could neglect the lateral dependence and considering only the longitudinal dependence on *z*. Furthermore by making the transverse wave approximation (*i.e.*, neglecting the **E** field projection in the direction of propagation), we get a more simple looking differential equation (*i.e.*, 1D plane wave approximation):

(6) $$\frac{\partial^{2}\text{E}}{\partial z^{2}}+\mu_{0}\varepsilon \omega^{2}\left(1+\frac{\Delta ∊(z)}{\varepsilon }\right)\text{E}=0$$

Equation (6) can be viewed as a scalar differential equation of the following type:

(7) $$\frac{\partial^{2}E}{\partial z^{2}}+\beta^{2}\left(1+\frac{\Delta ∊(z)}{\varepsilon }\right)E=0$$

Here $\beta =\omega \sqrt{\mu_{0}\varepsilon }$ is the propagation constant. Thus one could propose a solution composed of forward and backward traveling wave [10]:

(8) $$E=E_{0}e^{i\beta z}+\Psi (z,\beta )e^{-i\beta z}$$

The differential equation for a backward scattered wave is then:

(9) $$\frac{\partial^{2}\Psi }{\partial z^{2}}-2i\beta \frac{\partial \Psi }{\partial z}+\beta^{2}\frac{\Delta ∊(z)}{\varepsilon }E_{0}e^{2i\beta z}+\beta^{2}\frac{\Delta ∊(z)}{\varepsilon }\Psi =0$$

Considering the case of weak Rayleigh scattering by neglecting the second order derivative and the last term (*i.e.*,|Ψ| ≪ *E*_0_), we can find an approximate solution for the backward scattered Rayleigh light due to the random spatial variation of the permittivity:

(10) $$\Psi (z,\beta )-\Psi (0,\beta )\approx \frac{\beta E_{0}}{2i}\int_{0}^{z}\frac{\Delta ∊(\zeta )}{\varepsilon }e^{2i\beta \zeta }d\zeta$$

For most of the time the detected signal is Ψ(0, *β*) which is seen to be related to the end face reflection amplitude, Ψ(z = L, *β*). Hence the Rayleigh back scattered signal is a type of Fourier transform of the random permittivity fluctuation. Fiber attenuation *α* can be easily incorporated by the substitution of *β* → *β* + *iα*. The applications of Rayleigh scattering to the fiber sensing are relatively wide reaching; it can be used to sense local temperature or strain through detecting interference relative to a reference length. It can also be used to sense impact vibrations.

Although we emphasized that Rayleigh scattering is a type of scattering without frequency changes, *i.e.*, elastic scattering, there is other scattering arising from larger scattering centers such as dust particles giving no frequency change as well. Such processes are called Mie scattering, and their scattering strength is related to the size of the scattering particle and its refractive index relative to the scattering medium. Although Mie scattering can be used to detect dust particle size and it is widely used in biomedical sensing, while here we are more focused on scattering in fiber. The weak Rayleigh scattering refers to the comparison of pump signal; hence high order scattering can be neglected.

### 3.2. Spontaneous Brillouin Scattering in a Single-Mode Optical Fiber

For a perfectly symmetric waveguide, the fibre supports two orthogonally polarised modes, but they are degenerate. The radial intensity follows essentially a Gaussian distribution characterised by the spot size *r_a_* defined as the 1/*e* intensity width. The electric field propagating in the fibre is then considered as a plane wave with a Gaussian radial distribution.

The Brillouin scattering represents light scattering from the collective acoustic oscillations of the glass. From the microscopic point of view the intermolecular interaction in glass makes it favourable for molecules to stay at some stable distance away from each other. There is an energy penalty when the intermolecular distance is either farther apart or closer than this equilibrium position. This microscopic existence of balanced intermolecular distances leads to a new collective motion. Imagine if a neighbouring molecule was getting closer than the stable separation, it will then be pushed away towards the equilibrium point, however when it reaches the stable separation it will not stop, rather it will overshoot passing the equilibrium position, once it is farther away it will experience an attraction to pull it back toward the stable separation distance, however it will again overshoot when it returns. Such a repeating cycle forms a collective motion called acoustic phonons. To describe the above process we need to use macroscopic parameters like the density (***ρ***), entropy (***s***), pressure (***P***) and temperature of the matter. Recall these parameters are all macroscopic thermodynamic quantities that can be directly related to the macroscopic Maxwell equations. Assume material polarizability is proportional to material density if one analyzes the implications of the macroscopic Maxwell equation. Furthermore as the local density ***ρ*** is changed one can also expect local pressure changes as well. As we are interested in Δ*ε* variations induced by thermodynamic quantities, we first consider *ρ* and *T* as independent thermodynamic variables and write the dielectric constant as [15]:

(11) $$\Delta \varepsilon ={\left(\frac{\partial \varepsilon }{\partial \rho }\right)}_{T}\Delta \rho +{\left(\frac{\partial \varepsilon }{\partial T}\right)}_{\rho }\Delta T$$

According to [15], the second term can be neglected with an error of 2% because density fluctuations affect the dielectric constant significantly more than temperature fluctuations:

(12) $$\Delta \rho ={\left(\frac{\partial \rho }{\partial p}\right)}_{s}\Delta p+{\left(\frac{\partial \rho }{\partial s}\right)}_{p}\Delta s$$

The first term corresponds to adiabatic density fluctuations which is pressure waves or acoustic waves, *i.e.*, Brillouin scattering. The second term is entropy or temperature fluctuations, *i.e.*, Rayleigh scattering.

The dielectric constant fluctuation density can be expressed as:

(13) $$\Delta \varepsilon ={\left(\frac{\partial \varepsilon }{\partial \rho }\right)}_{T}{\left(\frac{\partial \rho }{\partial p}\right)}_{s}\Delta p=\frac{\gamma_{e}}{\rho_{0}}{\left(\frac{\partial \rho }{\partial p}\right)}_{s}\Delta \tilde{p}$$

where the electrostriction constant *γ_e_*, is defined as $\gamma_{e}=\rho_{0}{\left(\frac{\partial \varepsilon }{\partial \rho }\right)}_{T}$ [15], *ρ*_0_ is the average density of the fiber material. The acoustic wave is captured in the following wave equation describing the pressure wave with the local pressure variation parameter, Δ *p̃*:

(14) $$\frac{\partial^{2}\Delta \tilde{p}}{\partial t^{2}}-\Gamma^{\prime }\nabla^{2}\frac{\partial \Delta \tilde{p}}{\partial \text{t}}-V_{a}^{2}\nabla^{2}\Delta \tilde{p}=0$$

where Γ′ is a damping parameter related to the local viscosity of the material while *V_a_* is the sound velocity. In solving the above equation as shown in [15], one can get the solution for Brillouin frequency Stokes and anti-Stokes waves as follows:

(15) $$\Omega_{B}≅\frac{2n(\omega )\omega }{\frac{c}{V_{a}}\mp n_{g}(\omega )}\approx 4\pi \frac{n(\omega )V_{a}}{\lambda }(1\pm n_{g}(\omega )\frac{V_{a}}{c})$$

where $n_{g}(\omega )=\frac{d[n(\omega )\omega ]}{d\omega }$ is the group refractive index, the upper sign is for anti-Stokes and the lower sign is for the Stokes side resonance respectively. For a given frequency of light its corresponding Stokes and anti-Stokes Brillouin resonance frequency difference is: $\delta \Omega_{B}=\Omega_{B}^{AS}-\Omega_{B}^{S}≅4n(\omega )\omega \cdot n_{g}(\omega ){\left(\frac{V_{a}}{c}\right)}^{2}$.

### 3.3. Birefringence Effect in Stimulated Brillouin Scattering (SBS)

When we consider the fiber birefringence and PMD effect [16], the stimulated Brillouin scattering process will become more complicated. Consider the simple case of two propagation constants in the fiber along the principal axes of ***x̂*** and ***ŷ***. Considering counter propagating beams in the fiber with positive *z* propagating light for the Stokes wave:

(16) $$\left|{\text{E}}_{1}(z,t)\rangle =E_{1x}\exp\left\{i[k_{1x}z-\omega_{1}t]\right\}\right|{\hat{\text{x}}}_{1}\rangle +E_{1y}\exp\left\{i\left[k_{1y}z-\omega_{1}t\right]\right\}|{\hat{\text{y}}}_{1}\rangle$$

For the pump wave propagating in the negative z direction we have:

(17) $$\left|{\text{E}}_{2}(z,t)\rangle =E_{2x}\exp\left\{i[-k_{2x}z-\omega_{2}t]\right\}\right|{\hat{\text{x}}}_{2}\rangle +E_{2y}\exp\left\{i\left[-k_{2y}z-\omega_{2}t\right]\right\}|{\hat{\text{y}}}_{2}\rangle$$

The beating via electrostriction in the fiber due to above two waves can be written explicitly [17]:

(18) $$\langle {\text{E}}_{2}(z,\text{t})|{\text{E}}_{1}(z,\text{t})\rangle ={\text{E}}_{1\text{x}}{\text{E}}_{2\text{x}}^{*}\exp\{\text{i}[({\text{k}}_{1\text{x}}+{\text{k}}_{2\text{x}})z-(\omega_{1}-\omega_{2})\text{t}]\}\langle {\hat{\text{x}}}_{2}|{\hat{\text{x}}}_{1}\rangle +{\text{E}}_{1\text{y}}{\text{E}}_{2\text{y}}^{*}\exp\{\text{i}[({\text{k}}_{1\text{y}}+{\text{k}}_{2\text{y}})z-(\omega_{1}-\omega_{2})\text{t}]\}\langle {\hat{\text{y}}}_{2}|{\hat{\text{y}}}_{1}\rangle +{\text{E}}_{1\text{x}}{\text{E}}_{2\text{y}}^{*}\exp\{\text{i}[({\text{k}}_{1\text{y}}+{\text{k}}_{2\text{y}})z-(\omega_{1}-\omega_{2})\text{t}]\}\langle {\hat{\text{y}}}_{2}|{\hat{\text{x}}}_{1}\rangle +{\text{E}}_{1\text{y}}{\text{E}}_{2\text{x}}^{*}\exp\{\text{i}[({\text{k}}_{1\text{y}}+{\text{k}}_{2\text{x}})z-(\omega_{1}-\omega_{2})\text{t}]\}\langle {\hat{\text{x}}}_{2}|{\hat{\text{y}}}_{1}\rangle$$

#### (a) For linear and circular birefringence

Under such a condition, the 3rd and 4th terms in the last equation will be zero (assuming zero dispersion for both principal polarizations), *i.e.*, 〈**ŷ**_2_∣**x̂**_1_〉 = 0 = 〈**x̂**_2_∣**ŷ**_1_〉. Each principal axis component beats with its corresponding counter propagating beam to excite two moving acoustic waves with frequency Δ*ω* = *ω*_1_ − *ω*_2_, and their corresponding momentum vectors are different due to birefringence, but the two principal axes cannot be resonant simultaneously with acoustic phonons due to their mismatched phase condition. Hence a quasi-resonance could be reached between values of 2n_x_*ω*V_A_/c and 2n_y_*ω*V_A_/c. There exists a pattern of slowly varying amplitude modulation via spatial beat length [17]:

(19) $${\text{L}}_{\text{B}}=\frac{2\pi }{\left|\left({\text{k}}_{1\text{x}}+{\text{k}}_{2\text{x}})-({\text{k}}_{1\text{y}}+{\text{k}}_{2\text{y}}\right)\right|}$$

#### (b) For elliptical birefringence in fibers

This is the most general case. This has a consequence in elliptical birefringence, *i.e.*, 〈**x̂**_2_∣**x̂**_1_〉 ≠ 1 ≠ 〈**ŷ**_2_∣**ŷ**_1_〉, and furthermore we have 〈**ŷ**_2_∣**x̂**_1_〉 ≠ 0 ≠ 〈**x̂**_2_∣**ŷ**_1_〉. There are four acoustic moving waves to be excited.

Taking the steady state approximation for the zero birefringence case, the complex acoustic field amplitude can be written as [15]:

$$\Delta \rho =\frac{\gamma_{\text{e}}{\text{q}}^{2}}{\Omega_{\text{B}}^{2}-\Omega^{2}-\text{i}\Gamma_{\text{B}}\Omega }\langle {\text{E}}_{2}(z,\text{t})|{\text{E}}_{1}(z,\text{t})\rangle$$

To include birefringence of the fiber in above equation, we assume the *principal axes remain unchanged along the fiber*, and use Equation (19), Brillouin resonance frequency associated with the principal birefringence axes of pump and probe waves in the fibers [17]:

(20a) $$\Omega_{\text{Bxx}}=\frac{V_{A}}{c}[n_{1x}\omega_{1}+n_{2x}\omega_{2}]$$

(20b) $$\Omega_{\text{Byy}}=\frac{V_{A}}{c}[n_{1y}\omega_{1}+n_{2y}\omega_{2}]$$

(20c) $$\Omega_{\text{Bxy}}=\frac{V_{A}}{c}[n_{1x}\omega_{1}+n_{2y}\omega_{2}]$$

(20d) $$\Omega_{\text{Byx}}=\frac{V_{A}}{c}[n_{1y}\omega_{1}+n_{2x}\omega_{2}]$$

**Figure 3.**
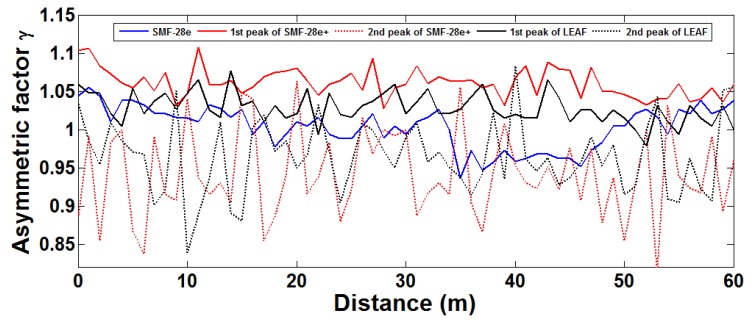
Asymmetric Brillouin spectrum property of SMF28e, SMF28e+ and LEAF *vs.* position.

It is important to comment that Equations (20c) and (20d) describe the cross polarization Brillouin resonance that can only exist in the event that the principal polarization states are elliptical in nature. In this case due to the counter-propagating nature between pump and probe light beams, a beating pattern could form between cross polarizations and thus resonate with an acoustic wave. Due to the local refractive index change associated with density fluctuation the SOP (state of polarization) of the pump and probe waves vary at different locations, so that the shape of the Brillouin spectrum changes due to the relative polarization orientation of the pump and probe wave and their overlap with “locally excited acoustic wave”. Hence the compound Brillouin frequency peak position is moved depending on detailed local SOP composition as described in Equation (20). Such a process will result in an asymmetric Brillouin gain spectrum at lower Brillouin gain. When the pump and probe waves take the **x** (Equation (20a)) or **y** (Equation (20b)) polarization, the Brillouin gain would be maximum and the respective gain spectrum tends to be symmetric. The asymmetric Brillouin gain spectrum can be observed in large effective area fiber (LEAF), SMF28e and SMF28e + fiber (fiber information can be found from data sheet of Corning). If we define asymmetric factor as $AF=\frac{\Delta \upsilon_{BR}}{\Delta \upsilon_{BL}},\Delta \upsilon_{BR}$ is the right spectral width from peak gain at half gain location. *υ_BL_* is the left spectral width from peak gain at half gain location. We can measure this asymmetric factor in experiment, which is conducted with a 50 ns pulse using 60 m of three different fibers with 40 different input SOPs. *AF* varies with position, and at the same position, it changes with different SOPs [17] as shown in Figure 3.

Conventional wisdom is to introduce a polarization scrambler (PS) to average many measurements based on different input SOPs in order to remove fluctuations in the polarization dependent gain and the Brillouin frequency, which will minimize the impact of different states of polarization on temperature and strain resolution of BOTDA.

Although introduction of PS can remove *AF* changes in the same position provided the pulse width is extremely small, it does not remove changes with position z. This change is introduced by the local acoustic speed fluctuation within the spatial distance covered by the pulse width in the optical fiber. Table 1 gives the statistics of measured Brillouin frequency fitted by Lorentzian function with SOP1 and SOP2 for maximum and minimum Brillouin gain, and with PS based on Figure 3 [18]. This means in distributed fiber Brillouin sensors using practical PS with non-ideal polarization scrambling, even if the measurement uncertainty of Brillouin frequency is reduced to a very low level (±0.016 MHz through averaging 40 repeated measurements), the accuracy of the detected Brillouin frequency shift is limited by the fluctuation caused by fiber inhomogeneity.

In BOTDA configuration, the SBS process will induce a nonlinear refractive index by pump and probe waves. Because this modulated refractive index is not purely from fiber birefringence as described by Equation (20), PS will not be able to completely remove its polarization dependence, *i.e.*, in BOTDA configuration the nonlinear interaction of the light fields with fiber would essentially make the PS not 100% effective, therefore the usage of practical PS can reduce but not eliminate the fluctuation caused by fiber inhomogeneity. As a comparison, under Brillouin optical time domain reflectometry (BOTDR) configuration, the refractive index change from SBS can be neglected, *i.e.*, PS is more effective to reduce the fluctuation caused by fiber inhomogeneity, while in this case the weak spontaneous Brillouin scattering leads to low signal to noise ratio (SNR) of the system.

For BOTDA sensor systems the ultimate temperature or strain resolution is limited by the maximum contribution of SNR of sensor system and inhomogeneity of the fiber, which has induced a range of the Brillouin frequency as described in Equation (20). Thus, simple index profile fiber is the preferable choice for distributed sensors.

### 3.4. Spontaneous Raman Scattering

When light is scattered from an atom or molecule, most photons are elastically scattered (local inhomogeneity would result in finite scattered light in directions other than original incident direction, *i.e.*, Rayleigh scattering), such that the scattered photons have the same energy (frequency) and wavelength as the incident photons. However, a small fraction of the scattered light (approximately 1 in 10 million photons) is scattered by an excitation, with the scattered photons having a frequency different from, the incident photons. The interaction of light with matter in a linear regime allows the absorption and emission of a photon precisely matching the difference in energy levels of the interacting electron or electrons.

In the Quantum Mechanics (QM) domain, a harmonic oscillator oscillates at an angular frequency *ω_M_* with quantized energy levels:

(21) $$E_{n}=\left(n+\frac{1}{2}\right)\hbar \omega_{M},n=0,1,2,\ldots$$

According to the statistical mechanics if such a quantum oscillator is in contact with a thermal reservoir of temperature *T* then this oscillator has probability *P_n_* being in the energy level *E_n_* given by:

(22) $$P_{n}=\frac{\exp\left(-\frac{\left(n+\frac{1}{2}\right)\hbar \omega_{M}}{k_{B}T}\right)}{\sum_{n^{\prime }=0}^{\infty }\text{exp}\left(-\frac{\left(n^{\prime }+\frac{1}{2}\right)\hbar \omega_{M}}{k_{B}T}\right)}$$

Here *k_B_* is the Boltzmann constant, and *ħ* = *h*/2π with *h* to be the Planck constant. Furthermore according to the QM the dipole transition strength from energy level *E_n_* → *E_n_*_+1_ is found proportional to quantum number *n* in the following: ${\left|\text{p}\right|}_{n,n+1}\propto \sqrt{n+1}$. Now we can evaluate the Stokes line strength from an ensemble of *N* identical oscillators connected to a thermal bath at temperature *T* [19]:

(23) $$N\sum_{n=0}^{\infty }{\left(\sqrt{n+1}\right)}^{2}P_{n}=\frac{\text{N}}{1-\exp\left(-\frac{\hbar \omega_{M}}{k_{B}T}\right)}$$

Conversely, the dipole transition strength from energy level *E_n_*_+1_ → *E_n_* is found in the following proportion ${\left|\text{p}\right|}_{n+1.n}\propto \sqrt{n}$. For the anti-Stokes line strength of an ensemble of *N* identical oscillators:

(24) $$N\sum_{n=0}^{\infty }{\left(\sqrt{n}\right)}^{2}P_{n}=\frac{\text{N}}{\exp\left(\frac{\hbar \omega_{M}}{k_{B}T}\right)-1}$$

We could write down the strength of the Raman Stokes line *λ_S_* from an ensemble of identical QM oscillator *ω_M_* that is dominated by the induced electric dipole radiation:

(25) $$I_{S}=I_{0}{\left(\frac{\ell }{\lambda_{S}}\right)}^{4}\frac{1}{1-\exp\left(-\frac{\hbar \omega_{M}}{k_{B}T}\right)}$$

Here *ℓ* is a length scale and *I*_0_ an intensity scale proportional to the incident light strength. At the same time the associated anti-Stokes Raman line *λ_AS_* has the strength:

(26) $$I_{AS}=I_{0}{\left(\frac{\ell }{\lambda_{AS}}\right)}^{4}\frac{1}{\exp\left(\frac{\hbar \omega_{M}}{k_{B}T}\right)-1}$$

We thus can derive the well-known expression that served as the basis for the distributed spontaneous Raman temperature sensor:

(27) $$\frac{I_{AS}}{I_{S}}={\left(\frac{\lambda_{S}}{\lambda_{AS}}\right)}^{4}\exp\left(-\frac{\hbar \omega_{M}}{k_{B}T}\right)$$

The above formula is derived with the assumption that each molecule is independent in the system and their mutual interactions are only represented by a statistical temperature parameter *T*. The first distributed Raman scattering sensor is based on above relation [4,6]. This technology has been widely used for temperature monitoring in oil well and energy pipelines [20]. The highest spatial resolution for Raman OTDR is 0.24 m with a sensing length of 135 m and temperature resolution of 2.5 °C [21]. The limited sensing length is due to the weak anti-Stokes Raman signal, 20–30 dB weaker than that of the Rayleigh scattering light, unless Raman gain is implemented to enhance the sensing length.

## 4. Application and Limitations of Stokes and Anti-Stokes Ratio Based Raman OTDR

In the early demonstration of Stokes and anti-Stokes ratio to measure the temperature with Raman scattering, the difference of the fiber attenuation at the Stokes line *λ_S_* and anti-Stokes line *λ_AS_* were not counted. In reality, because of the large wavelength difference of Stokes and anti-Stokes lines, it can be 200 nm or larger at 1,550 nm, depending on the type of the fiber; typically, single mode fiber has attenuation of 0.2 dB/km at 1,550 nm, and 0.4 dB/km at 1,310 nm. The temperature difference of the Stokes and anti-Stokes ratio is comparable to the fiber loss difference at two wavelengths, which is 0.8%/°C at room temperature in SMF-28 fiber [4,6]. Hence, a few methods have been proposed to automatically correct this error.

In the spontaneous Raman scattering distributed fiber temperature sensor one necessarily uses a high intensity optical pulse to induce the reflected Stokes and anti-Stokes signal to be detected. The reflected Stokes and anti-Stokes light pass through the same fiber length with different attenuation. If one were simply using their reflected ratio to decode the temperature without this attenuation difference, it would introduce error. This can be corrected by using the dual end method [22], due to the variance of attenuation between the Stokes and anti-Stokes lines for the entire fiber length. The disadvantage of the dual end method is that it requires access to both fiber ends at the same time, which would essentially reduce the usable fiber sensing length to half.

Another method is the double light source method [23,24], which uses two different wavelengths to measure the loss ratio of Stokes and anti-Stokes signal. Compared with the dual end method this double light source method is in principle more robust as it achieves the exact cancellation for the attenuation factors with two specific wavelengths, but it requires wavelength stability of the two light sources, hence an extra expense for practical application. However it does offer higher precision for better temperature resolution.

Both the dual end and double light source methods need to measure both Stokes and anti-Stokes lines, an alternative solution is to use a single light source to measure either Stokes or anti-Stokes lines instead of two lines [25,26].

The range of the Raman OTDR sensors is typically limited to about 10 km. This sensing length limitation is due in large part to fiber loss and fiber intermodal dispersion (if multi-mode fiber is used as the sensing fiber). Another factor affecting the OTDR range is the repetition rate of the pump laser, but this can be modified with modulation, either internal or external to the pump laser. The spatial resolution of the OTDR is determined by the convolution of the laser's pulse width with the response function of the detection system.

For this reason, high powered lasers and long acquisition times are needed; therefore, early work employed multi-mode fibers to increase the collection of backscattered photons, and, pump lasers with wavelengths near 800 nm–900 nm were chosen so that high performance silicon avalanche photodiodes (APDs) could be used for detection as first demonstrated [27], and 50 to 125 μm GRIN (gradient index) fiber must be used to avoid excessive signal averaging time.

However, fiber intermodal dispersion in MM fiber has limited spatial resolution as well as sensing length, as MMF has higher attenuation, when telecom-grade low-loss single-mode fiber is implemented, a measurement distance range of 40 km in single-mode dispersion shifted fiber, and using optical amplification in combination with coded pulses has been demonstrated [28]. Although sensing length has been extended significantly, the temperature resolution has been reduced to 5 K, and the spatial resolution increased substantially to 17 m. Such a performance is far from what can be achieved with Brillouin scattering based distributed sensors, in which a sensing length of 150 km and spatial resolution of 2 m with 2 °C temperature resolution has been demonstrated over a measurement time of a few minutes [2]. Currently available commercial systems of Raman OTDR provide resolutions of 5 m for distances up to 30 km. Measurement times are on the order of minutes [29].

The best spatial resolution is achieved with a multi-photon counting technique to give resolutions of a few to tens of centimeters [21,30] over a few meters of sensing length. Most recently, a single photon-counting technique has been introduced in a Raman OTDR system to improve its low spatial resolution. Because of the high detection sensitivity required for photon counting; spatial resolution on the order of 1 cm at 1,550 nm wavelength in a single-mode fiber was realized using superconducting nanowire single-photon detectors [31]. In this recent demonstration a rapid measurements based on a 60 s integration period and temperature uncertainty on the order of 3 °C with only a 3 m fiber length has been demonstrated. This is a significant improvement in terms of spatial resolution. However, it is still rather far from practical application, especially considering the advancement of Brillouin scattering based technology, which can obtain 2 cm spatial resolution with a sensing length of 2 km and temperature resolution of 2 °C [32].

The applications of distributed temperature sensors includes the power supply industry, in which fiber sensors are inserted into high power transformers to detect hot spots and determine their temperature. As the highest temperature determines the lifetime of the insulation materials, so does the equipment itself. By continuously monitoring the hot spots, failure of the transformers can be prevented. They can also be used in thermal power stations to monitor high pressure steam pipes for leaks [33], and for pipeline temperature monitoring to search for leaks in fluids, because cooling will be generated from the expansion of gas leaving the pipes. Another use is for the monitoring of heating materials to reduce viscosity.

Storage vessels in industry require leak monitoring, this is particularly true for liquefied natural gas, and hence an optical fiber can be used to monitor the temperature change. More applications can be found in the processing industry to monitor long term thermal curing or drying processes as well as fire alarms in tunnels and buildings.

## 5. Rayleigh Scattering Based OTDR

OTDR was first introduced to monitor fiber attenuation [3] for fault detection in telecommunication cables, later such systems have been used for a number of other applications. These all involve enhancing the effects of the measurand on the loss of the fiber, thereby allowing that measurand to be profiled along the fiber length. One source of loss is micro-bending and a distributed sensor system to measure lateral pressure on a fiber has been developed [34] that places a fiber inside a spiral sheath that induces micro-bending in the fiber when a lateral force is applied. The original idea was developed for mechanical sensing based on micro-bending induced loss from small and sharp bends on fiber [35] which increase local attenuation using single mode fiber. The extra loss is detected through standard OTDR techniques, where the system is probed with narrow pulses following an optical radar concept. Rayleigh back scatter from the fiber material can be detected, and changes can be seen in different medium properties such as discontinuities in the fiber or coupler. Ultimately spatial information is acquired by mapping position to the time of flight of a pulse of light traveling to and from the sensing location. There are three types of Rayleigh OTDR systems.

### 5.1. Conventional OTDR

Broadband source based OTDR is commonly used in conventional OTDR instrumentation as described above. A Rayleigh scattering based temperature sensor is possible, at least in principle, through the dependence of the Rayleigh scattering intensity on temperature. For normal glass fibers the dependence is too weak to result in an effective sensor; however a successful approach is to use liquid core fibers to measure temperature with an accuracy of ±1 °C with a spatial resolution of a few meters [33]. Due to a weak Rayleigh signal, high powered lasers and long acquisition times are needed, and the fiber was a silica glass tube filled with a higher-refractive index low absorption liquid, which plays the role of the light-guiding core of the waveguide. The scattering loss coefficient of the liquid depends on the density fluctuations caused by thermodynamic molecular motion; hence it has strong temperature dependence. The practical application of this sensor is limited by the need for special liquid core fibers.

The use of temperature variation of the attenuation coefficient of doped glass fibers has been studied. These sensors rely on the absorption bands of the dopants which shift with temperature. By monitoring the loss near the edge of an absorption band, changes with temperature can be seen as changes in attenuation. Results obtained with Nd doped fiber showed 2 °C accuracy and 15 m resolution over a 200 m sensing length [36]. Using Ho-doped fiber these figures were improved to 1 °C and 3.5 m [37]. Many of the absorption bands result from transitions between electronic levels that are temperature dependent, which means that their attenuation coefficients are temperature dependent, making them potentially useful for distributed temperature sensing. Taking advantage of this behavior also comes with a disadvantage, as the temperature dependent coefficient often has much higher loss than conventional SMF; the sensing length becomes significantly limited. If one can find a candidate with low loss and high temperature coefficient, and then it would be an attractive approach for practical applications. At present, OTDR is most generally applied by the communication industry to find high loss fiber links.

### 5.2. Polarization OTDR

POTDR uses a broadband frequency laser of ∼0.1 nm to create a polarized pulse of light. The fiber loss is modulated by the local polarization state change and is detected by backward Rayleigh scattering. If any disturbance occurs in one location, the SOP will be modulated. Based on this approach, polarization OTDR was proposed and demonstrated [38,39], and it monitors the spatial distribution of the fiber's polarization properties which can be modulated by pressure, strain, temperature, as well as electrical and magnetic fields. Because polarization changes are induced by various parameters, it is difficult to differentiate the contributions of individual parameters.

For static processes POTDR can be used to measure bend-induced birefringence in single-mode fibers [40], and twist induced birefringence in short lengths of fibers [41]. It can also be used to extract the intrinsic local birefringence in a single-mode fiber [42,43]. The beat length is inversely related to the birefringence, defined as the phase delay difference between the two principal states. Using POTDR one can measure distributed beat length [43, 44] by trace analysis through computation of the spatial distribution of birefringence. This has opened a door through birefringence measurement to obtain PMD from beat length, because the PMD of a fiber is related to the mean beat length and coupling length.

Before PMD is measured by the polarization analyzer [42], a four-channel receiver is needed to record simultaneously the four signals provided by the analyzer, so that PMD can be measured rapidly. The POTDR requires three input polarization states and a computational technique to recover PMD.

PMD is a major limiting factor for high speed fiber communication system because of its induced pulse broadening, and it is very difficult to be compensated since PMD changes with time [45]. Normally high PMD occurs in one or two sections of the fiber either due to the fiber manufacturing process or local environmental changes, such as high temperature gradient, strong wind, or sun radiation induced temperature gradients on the cabled fiber [46]. It is important to identify and locate the high PMD section through spatial distribution of the fiber's polarization properties. Using POTDR, such a task has been realized by a few groups [44,47,48], the techniques are based on a measurement of the degree of polarization of the backscattered light as a function of distance in the fiber. Both the average and statistics of the degree of polarization are used to estimate the beat length and the coupling length, with a review paper [49] providing details of spatially resolved PMD and PDL measurement using POTDR.

The drawback of POTDR for purposes of static process sensing is the variety of parameters that can impact the polarization state change, such as temperature and strain. As a result, the fiber SOP appears to drift by itself with time, however such changes typically only occur over periods of few minutes [45], hence dynamic measurements can be made with POTDR for vibration sensing such as a distributed vibration sensor with 2 km sensing length, 10 m spatial resolution in which 5 kHz vibration frequency and double events were detected [50]. Because the SOP change in one position will affect the following position, this induces a significant location uncertainty, even for the dynamic measurement of vibration. Generally, POTDRs are mostly used in the communication field for locating fiber sections with high PMD values in the manufacturing plants and fiber networks.

### 5.3. Coherent OTDR and Phase OTDR

Coherent OTDR means low coherent OTDR using coherent detection [51]. Coherent detection is realized by optical mixing of the backscattered light and reference light. With the balanced detection technique, the DC noise is reduced significantly, which gives a shot-noise limited sensitivity of −140 dB at a 3 Hz bandwidth for millimeter spatial resolution when the sensing length is reduced to about meters, in which photon counting technique is introduced [52].

Phase OTDR means coherent light source with direct detection [53]. In this case, a kHz linewidth laser is used with short pulses for coherent detection or large pulses for direct detection, where the spatial resolution of a few hundred meters can be achieved with 12 km of fiber for intrusion sensing. Because of coherent Rayleigh scattering accurate location information can be identified, unlike polarization OTDR which can only be used as an alarm system for locating a starting point without its ending point, due to the continuous SOP change in the optical fiber from the disturbance.

With coherent detection of phase OTDR [54], 2 km sensing length has been demonstrated with 10 m spatial resolution and 1 kHz vibration frequency [55,56] using SMF28 fiber. The key to the location of a high frequency event is to use an optical wavelet technique to remove the birefringence noise. By changing to polarization maintaining fiber (PMF), the spatial resolution has been improved to 1 m, and the frequency range has been increased to over 2 kHz, furthermore, the disturbance point can be as far as 18 cm from the detection point with a metal plate acting as a transducer to cover the area [56]. Further improvement is obtained by a wavelet de-noising technique in coherent detection of phase OTDR in SMF-28, the spatial resolution has been reduced to 50cm and the vibration frequency has been increased to 8 kHz over a 1 km sensing length [56], and the event can be located as far as 20 cm from the sensing fiber.

In general, the spatial resolution in OTDR based sensor systems is determined by the pulse width in the optical domain, while in the electronic and digital domains, it is determined by the bandwidth of the detector, electrical amplifier and digitizer. With high precision spatial resolution, on the order of a millimeter, the required bandwidth is in the range of tens of GHz, which makes the distributed sensor system very expensive and complicated. One alternative solution is the OFDR [10], which will be explained in the following section.

## 6. Rayleigh Scattering Based OFDR

**Figure 4.**
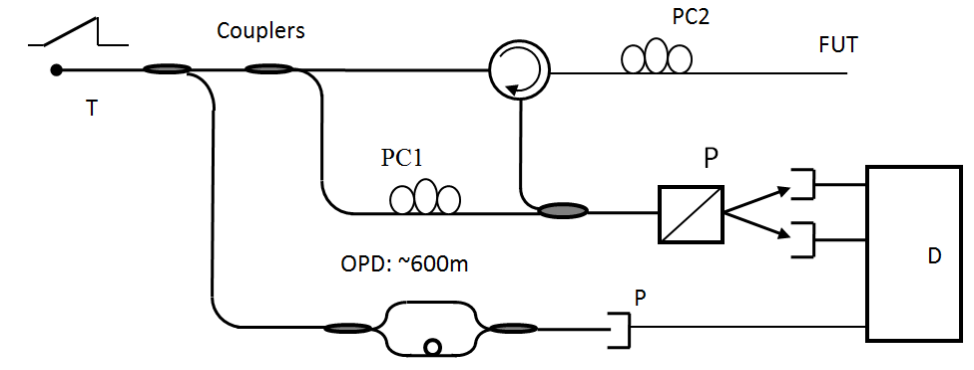
Experimental setup of OFDR.

The drive for short spatial resolutions of millimeter scale and cost effective distributed fiber sensors has pushed the interest in OFDR systems. Because a mm equivalent spatial resolution in OTDR systems would require a data acquisition card with a bandwidth of 10 GHz and a sampling rate of tens of GSamples/s, such a digitizer or data acquisition card plus the pulse generator and detection system will make a distributed sensor set very expensive. While the spatial resolution of the OFDR sensor system tunes a frequency range and converts the frequency response of the fiber into the time/spatial domain by Fourier transform, the spatial resolution does not depend on bandwidth of the detector or digitizer, rather the frequency tuning range of a tunable laser. It offers an alternative solution for a high spatial resolution sensor system. Due to the large scanned frequency range at high power density OFDR has the potential to achieve high spatial resolution. The typical OFDR layout is shown in Figure 4.

Although Rayleigh scattering itself is independent of temperature and strain as explained in Section 3.1, through optical coherent detection as illustrated by Figure 4, a change of sensing length (fiber under test) relative to the fixed reference length of local oscillator in the interferometer provides temperature and strain change induced phase differences via index variation, which can be measured at each fiber location [57]. As temperature, vibration and pressure changes will induce index variation, which can be measured by phase detection from interferometer. Hence, OFDR can be used for temperature [58,59], and strain [10] sensing by measuring the path length difference of the Rayleigh scattered light and a reference arm for their physical length change and index change.

As the Rayleigh scattering in an optical fiber is caused by fluctuation of the dielectric constant and is associated with the thermodynamic change of density and temperature, when a polarized light is launched into a fiber, the state of polarization (SOP) of the Rayleigh scattering changes continuously along the fiber, requiring a detection system with a polarization diversity (PD) scheme to remove the SOP dependence. The vector sum of ***p*** and ***s*** components can be calculated for the fiber without strain or temperature and then re-calculated with the strain or temperature applied. The two profiles are then separated into many segments of ***Δs*** for performing Fourier transforms to the frequency domain. A cross-correlation is performed for each segment to determine the spectral shift between the reference and perturbed profiles. The spectral difference between the shifted peak and un-shifted peak is directly proportional to the temperature or strain changes in each segment.

Nonlinearity of the tunable laser is corrected by an unbalanced auxiliary Mach-Zehnder interferometer (AMZI), which used as a trigger signal, can sample the Rayleigh scattering signal. The maximum measurement length *L*_max_ is approximately determined by the Nyquist sampling criteria to be:

(28) $$L_{\text{max}}=\overset{c\tau_{g}}{\;}/\underset{4n}{\;}$$

where τ_g_ is the differential delay in the auxiliary interferometer used to calibrate the tunable laser induced nonlinear wavelength sweep. The factor of four in the denominator is due to the sampling theorem and the double-pass nature of the measurement interferometer. The coherence length of the light source also limits the maximum measurement range. Using a phase-reconstruction scheme the maximum length can be improved to 1 km. The recovered temperature and strain information through *auto- and cross-correlation* in PMF can be obtained simultaneously, because two axes have different temperature and strain coefficients [60]. This means that using PMF one can get simultaneous temperature and strain sensing, so long as the position dependent birefringence change due to temperature and strain can be neglected, and calibration is crucial for different kinds of PMFs.

The spatial resolution of the measurement, *Δz*, is directly related to the resolution in the time domain and is determined by the optical frequency sweep range ΔF as follows:

(29) $$\Delta z=\frac{c}{2n_{g}\Delta F}$$

The preceding equation represents the theoretical resolution associated with the total optical frequency span corresponding to the measurement, in practice, this resolution can be affected by environmental noise, insufficient linear laser tuning and an excess of unbalanced dispersion in the measurement arm. Simultaneous temperature and strain measurement can also be realized with plastic optical fiber for a 2 cm spatial resolution with a potential 70 m range and strain and temperature resolution better than 35 με and 3.5 °C [60].

The OFDR technique is well suited for the purpose of optical network components characterization with millimeter spatial resolution, as well as in civil structural monitoring of micro-crack formation. One of the early applications is local birefringence measurements [61]. The measurement was taken by counting the coupling length between two axes in the reflection spectrum versus position. For the case of a coupling length *h* (*h* ≈ 12 *m* for SMF-28 fiber) which is much smaller than the fiber length *l*, and using the approximation that the group birefringence (B) is equal to the phase birefringence (β = B), PMD can be calculated as [61]:

(30) $$\text{PMD}=\frac{\lambda }{CL_{B}}\sqrt{lh}$$

where *c* is speed of light in optical fiber, and *L_B_* is the beat length.

In addition to temperature, strain and beat length sensing, OFDR can also be used to detect high order mode coupling in tapered fibers by measuring the index difference between different modes. The relation between the wavelength shift and the refractive index difference at position z of different modes is obtained by [12]:

(31) $$\Delta n_{ij}=\frac{n_{i}}{\lambda_{i}}\Delta \lambda_{ij}$$

where *Δλ_ij_, λ_i_*, and *n_i_* are the wavelength difference between two modes which are measured by OFDR auto-correlation function at one location, wavelength, and refractive index for the *i*^th^ mode, respectively, inside the fiber, and *Δn_ij_* = |*n_i_-n_j_*|. Therefore, the refractive index difference between the fundamental mode and the high order modes of a segment of high order mode fibers can be obtained by measuring the wavelength shift using OFDR scheme.

OFDR technology is excellent for short sensing lengths (<100 m [58–60]), but longer reach is possible at the cost of spatial resolution, and temperature/strain resolution, as summarized below:

1. Spatial resolution is limited by tuning range and chromatic dispersion. Large tuning range ΔF corresponds to higher spatial resolution as seen in Equation (29), however with larger wavelength range varying group velocities result in larger spatial resolution due to chromatic dispersion of the fiber, because different frequency components travel at different speeds, while in ordinary FFT processing all the frequency components are treated as having the same speed.
2. Trigger interferometer induced limitation: When the sensing length is increased, the delay length of the trigger interferometer increases too, which is four times the sensing length. As local environmental conditions such as temperature and vibration change, there is an impact on the measured wavelength interval, which is treated as a “clock” to count the interval of the returned signal. When temperature or vibration changes, the “clock” becomes uneven, and since the data processing cannot correct this mistake, the spatial location will be smeared at the far end of the fiber due to accumulation of environmental effects on the delay length of the trigger interferometer.
3. Maximum sensing length is limited by phase noise of the laser. For a given reflection the phase noise increases with its distance to the local oscillator and is proportional to the reflected light intensity [62], so that not only does the signal intensity decays but the noise floor also increases with distance from the reflection site to the local oscillator. For long sensing lengths, the phase noise could be higher than the Rayleigh scattered signal, thereby setting the maximum detectable length.
4. Fading noise sets the detectable spatial resolution: The oscillating behavior of the backscattering signal, called fading noise, arises because of the interference of sinusoidal waveforms with random amplitudes, which are contributed by neighboring parts of certain sections of the test fiber. Because of this random character, the resulting total intensity fluctuates and provides the noisy feature of the reflectogram. This feature strongly restricts the possibility of detecting small reflections in the fiber because they are induced by the Rayleigh backscattering fluctuations [63]. Fading noise can be reduced significantly by signal processing after FFT via moving average.

The fiber length in OFDR can be improved if a single-side-band modulator and a narrow linewidth laser is used to replace a tuneable laser, a cm level resolution over 5 km measurement range with high sensitivity and a noise level 23 dB lower than the Rayleigh backscatter level was demonstrated to locate high loss points [64].

The strain or temperature measurement in Rayleigh scattering based OFDR gives relative measurements as it is based on path length differences of the interferometer at the same position under different strain or temperature conditions. If the change of temperature or strain between two measurements is larger than the correlation peak width, OFDR will give a zero correlation. For larger temperature or strain changes, a Brillouin scattering based sensor system is a better approach, as this type of the system uses the frequency shift of the Brillouin peak, which is an absolute measurement based on the density variation induced sound velocity change as explained in Section 3. In addition, Brillouin scattering based OTDR systems have much longer reached, over 100 km sensing length, which is essential for pipelines and larger civil structures.

Although the Rayleigh scattering signal is 15–20 dB stronger than that of Brillouin scattering, in Brillouin scattering based distributed sensors, often the Brillouin spectrum is measured with many averages for each frequency components over 80–100 frequency points. As peak fitting of the Brillouin spectrum gives the temperature and strain reading, the signal to noise ratio has been improved significantly through this process. Furthermore, frequency is an absolute measurement, while in phase OTDR the power is measured at one frequency to recover the external change, which is a relative measurement. Averaging the waveform helps to remove the polarization dependence and laser phase noise changes in each trace, which sets the limitation for spatial resolution of OTDR sensor. In OFDR no averaging can be taken, and spectrum recovery is realized over a wide wavelength range. As a result of the polarization and wavelength dependence, optical coherent detection of external changes, and laser phase drift, the sensing length and strain resolution is limited in such systems.

## 7. Brillouin Scattering Based Distributed Sensors

While there are a variety of distributed fiber optic sensors, research on distributed strain sensing has focused almost exclusively on Brillouin scattering based systems. For over two decades, distributed optical fiber sensors based on Brillouin scattering have gained much interest because of their potential for monitoring temperature and strain in large infrastructures, replacing thousands of point sensors due to their precision, long sensing length, and high spatial strain and temperature resolution. These kinds of sensors can find applications in civil structures, environmental monitoring, the aerospace industry, power generator monitoring and geotechnical engineering.

The principle of the distributed Brillouin scattering sensor for temperature measurement is different from that of the Raman scattering sensor, as temperature change modifies the mean density that is associated with the velocity of sound, it can influence mechanical waves. Although the effective refractive index also affects the change in Brillouin frequency variation via temperature and strain, the dominating effect of temperature and strain is on density (hence sound velocity), which has been derived in Section 3. While in Raman scattering, the temperature change induces the transition between rotation and vibration levels of molecules. Hence quantum mechanics was introduced in Section 3 to treat the energy transition. The spectrum in Raman scattering is on the THz scale, while the spectrum in Brillouin scattering is on the MHz scale. Hence the measurement in Brillouin based distributed sensors is usually focused on the frequency measurement, *i.e.*, to measure the temperature or strain change induced Brillouin peak frequency shift, while in Raman scattering based sensors, the measurement is related to the power measurement over a wide frequency range (THz).

### 7.1. Development of BOTDR and BOTDA

The first work leading directly to strain sensing based on Brillouin scattering was carried out in 1989 [65], in which the Brillouin shift of optical fiber was found to be linearly related to applied strain. The Brillouin shift was also found to be linearly related to temperature [66]. The first demonstration of Brillouin scattering spectrum in a distributed fashion was based on stimulated Brillouin scattering, so called Brillouin optical time domain analysis (BOTDA), which used two counter-propagating lasers and took advantage of Brillouin amplification. Distributed temperature measurement [67] with 3 °C temperature accuracy and a spatial resolution of 100 m over a sensing length of 1.2 km was demonstrated. Later, a BOTDR was proposed with the advantage of monitoring the system from one end of the sensing fiber [68]. Performance was improved by coherent detection with the total sensing length being increased to 11 km with similar spatial resolution and temperature accuracy.

For the BOTDA system, because it used pump and probe wave counter-propagating in a fiber, when the frequency difference between a pulsed pump *ν_0_* and continuous wave (CW) probe *ν_0_*-Ω*_B_* is matched with local Brillouin frequency Ω*_B_* as described in Equation (15), the probe wave signal will be amplified at this location. By scanning the probe wave frequency, one can obtain the calibration coefficients and peak frequency by fitting the Brillouin spectrum with a Lorentzian curve, whenever the temperature or strain is changed, the peak frequency will be shifted. The temperature and strain coefficient of a specific fiber can be measured by calibration coefficients as:

(32) $$\Delta \upsilon_{B}=C_{T}\Delta T+C_{∊}\Delta ∊$$

where *C_T_* (1.26 MHz/°C) and *C_ε_* (0.056 MHz/μ-strain) are temperature and strain coefficients for SMF-28, and they change slightly with different kinds of single mode fiber.

With the optimization of the pump depletion and probe wave saturation for more uniform Brillouin gain, a significant improvement in sensing length and spatial resolution have been reported -with accuracy of 1 °C for temperature measurements [69], having a spatial resolution of 10 m and a total sensing length of 22 km.

This performance was improved upon quickly by the introduction of the Brillouin loss BOTDA configuration, which also attained a temperature measurement accuracy of 1 °C with a spatial resolution of 5 m and a total length of 32 km [70]. Eventually, the total sensing length of this configuration was pushed to over 50 km [71]. In addition to working on extremely long distance temperature sensing, a strain measurement accuracy of 20 μm per meter fiber was also demonstrated with a spatial resolution of 5 m and a total sensing length of 22 km using the same Brillouin loss setup via the Brillouin loss mechanism, in which the pulsed signal is a Stokes wave, while the pump signal is a CW wave. With such a configuration, the CW wave as pump can provide significant gain for the pulsed Stokes wave, which acquires energy from the CW pump, and yet depletion of the pump can be neglected. The key to achieving long length fiber sensing is to limit the pump power, so that low gain can be maintained over the entire sensing length, preventing gain saturation of the Stokes wave, and reducing pump wave depletion. In addition to the power requirement, polarization state matching is also critical, as the choice of SOP for pump and probe waves should maintain modest gain over the entire sensing length, rather than high gain at the front of the fiber section. This is very different from the condition of short sensing length, in which SOP matching is required to have as much gain as possible. On the fiber side, high stimulated Brillouin threshold fiber is preferred for long sensing lengths to ensure low Brillouin gain over the entire fiber.

The simultaneous measurement of both strain and temperature using a single fiber [72] was also demonstrated by isolating half the length of the fiber from strain, so that it was only sensitive to temperature, while the remaining half was sensitive to both parameters. By laying the two sections of fiber in parallel, it was possible to separate the effects of both parameters by comparing the Brillouin shift occurring in each section of fiber. Accuracies of 20 μm per meter of fiber and 2 °C were obtained, with a spatial resolution of 5 m over a total fiber length of 22 km.

An alternative layout of BOTDA was to use an Electric Optical Modulator (EOM) to create a sideband of the Brillouin frequency. Compared to two laser systems, this setup added a micro-wave generator and broadband amplifier as EOM driver and EDFA. Hence the cost of this setup is comparable to the two laser system. The advantage of this technique is that it used only one laser, and took advantage of fiber amplifiers for both pump- and probe-waves [73]. This system has the performance of 45 m spatial resolution on 1.4 km sensing length. It was eventually developed to the point that one meter spatial resolution was attained [74]. At the time it was felt that 1 m spatial resolution was the limit of spatial resolution for Brillouin scattering based sensors due to the observed broadening of the spectral line width [74,75].

Landau-Placzek ratio was explored to be sensitive to temperature [76] and it could be combined with Brillouin frequency dependent temperature and strain to measure simultaneous temperature and strain. Due to the weak spontaneous Brillouin signal, Landau-Placzek ratio method could only get temperature accuracy of 10 °C with a spatial resolution of 600 m. Further development has improved the spatial resolution to 10 m, which could be used for some applications [77]. This technique has been refined with Raman fiber amplifiers and coherent detection, which allows 150 km sensing length [78] at the temperature resolution of 5.2 °C with a 50 m spatial resolution.

At the same time simultaneous temperature and strain measurements were also made by determining the temperature dependence of the spontaneous Brillouin signal in BOTDR [79] using a single fiber, achieving 100 με and 4 °C accuracy with a spatial resolution of 40 m and a total sensing length of 1.2 km. The measurement time was on the order of one hour, making it very impractical, but it did demonstrate that simultaneous measurement of temperature and strain was possible by observing only the Brillouin spectrum of a fiber. Later, a simultaneous measurement system using polarization maintaining sensing fiber with Brillouin gain of BOTDA was achieved with significantly better performance. A strain resolution of 128 μm per meter was obtained, with a temperature resolution of 3.9 °C at a spatial resolution of 3.5 m, an order of magnitude better than that by the spontaneous scattering method [80].

Because of the polarization state change induced optical power fluctuation, simultaneous temperature and strain sensing based on the power and Brillouin frequency tends to have low precision for temperature and strain measurement. Furthermore, the spatial resolution is usually in the range of a few meters, unless different technologies are combined, such as Brillouin optical correlation domain analysis (BOCDA) to measure the Brillouin frequency and anti-Stokes line of BOTDR [81] to measure the power change. In such systems temperature resolution of 2 °C simultaneously with strain resolution of 63 με and spatial resolution of 5 cm are demonstrated over 18 m. The setup is complicated with two detection systems for coherent (BOCDA) and direction detection for BOTDR.

The first demonstration of a Brillouin scattering sensor system with spatial resolution substantially better than one meter (50 cm) was presented in 1998 [82], and included the 1st structural monitoring of strain measurements rather than simply measuring stretched sections of fiber. This was further improved to 10 cm spatial resolution [83] by using a spectrum de-convolution technique; in which two pulses were sent with their trigger times delayed by ½ pulse, so that the recovered spatial resolution could be reduced by ½ of the pulse length in the spatial domain, and the same for the Brillouin spectrum domain to recover different strain components.

### 7.2. Frequency Domain Distributed Brillouin Sensor

A Brillouin Optical Frequency Domain Analysis (BOFDA) was developed to measure the complex baseband transfer function by the ratio of the Fourier transforms of the pump and Stokes intensities [84]. Then the inverse-Fourier transform is taken to give the temporal pulse response which can be converted to a spatial response by using the relationship *t* = *2nz/c*. The intensities of the two beams are detected and fed into a network analyzer which determines the baseband transfer function. This in turn is fed into a digital signal processor which takes the inverse fast Fourier transform (IFFT), giving the pulse response of the fiber at the given laser beat frequency. Initial results performed using this system showed an accuracy of 1.5 °C and 40 μm length change per meter fiber with 1.4 m spatial resolution on an 11 km long fiber.

An application of the BOFDA sensor has been reported for beat length measurements by locating the areas of maximum and minimum Brillouin loss. This was done using the BOFDA method and beat lengths in the range of 50 to 60 m were observed on a 10 km length of fiber [85].

Great effort has been put towards getting centimeter spatial resolution with BOFDA technology; post-signal processing is critical in getting small stress or temperature resolution. 3 cm spatial resolution has been demonstrated with 9m sensing length and the resolution for the Brillouin frequency shift after de-convolution is 1.8 MHz. This is equivalent to 1.8 °C and over 30 με [86].

Another frequency domain BOTDA system is the Brillouin Optical Correlation-Domain Analysis (BOCDA) [87]. The spatial resolution of the BOCDA system is determined by the modulation parameters (amplitude and frequency) of a light source, rather than by the decay time of an acoustic wave. In this approach, two CW light waves with a Brillouin frequency difference are identically frequency-modulated. SBS occurs at the correlation peak position, where the two lightwaves are highly correlated. The correlation peak width determines the spatial resolution. Signal processing of the correlation between different positions provides a sharp peak for the matched Brillouin frequency, and the spatial resolution of BOCDA can be as high as 1cm for short sensing lengths of tens of meters [88]. The most recent report has improved this sensor system to 1 km length of 7 cm spatial resolution [89].

### 7.3. Differential Brillouin Gain Based on Differential Pulse-Width pair (DPP-BOTDA)

When the pulse was reduced to 1 ns, which is equivalent to 10 cm, the weak Brillouin signal due to short interaction has reduced the signal to noise ratio significantly, on top of that the broadband frequency of a short pulse requires a broadband detector and electrical amplifier, which adds significant electronic noise to the signal. As a result, the achievable strain or temperature resolution has been decreased significantly. Although pre-pumping [90] through a DC portion of the pulse could amplify the Brillouin signal, it does not help to improve the contrast of the Brillouin gain signal, as it adds a constant temperature fiber portion as background for the signal. To solve this problem, differential Brillouin gain detection using two pulses with slight pulse width difference was proposed [91], so that the spatial resolution can be determined as the pulse width difference instead of the pulse itself. In this way the large pulse reduced the low Brillouin gain signal due to the impact of the phonon lifetime (10 ns), and at the same time the narrow Brillouin gain spectrum could be obtained due to the large pulse. As a result, the signal to noise ratio is improved, especially on the temperature or strain resolution due to much narrower Brillouin spectral width.

**Figure 5.**
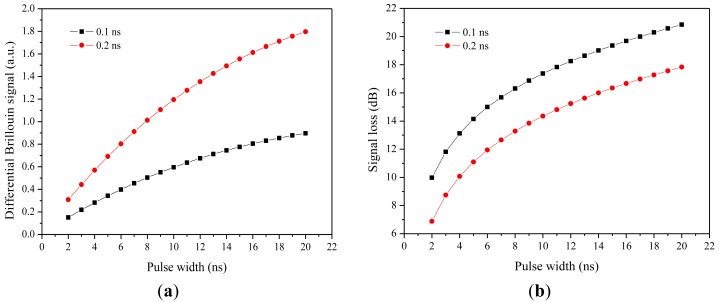
(**a**) Differential Brillouin signal (intensity) [32]; and (**b**) Brillouin signal loss as a function of pulse width (here refers to the shorter pulse of the pulse pair) for 0.1 and 0.2 ns pulse width difference.

The measurement is conducted in two consecutive waveforms with different pulse width. As a result, the problems of Brillouin spectrum broadening and small interaction length could be solved and higher gain is expected due to the last portion of the pulse being used for the pulse width difference. The signal loss in differential Brillouin gain and Brillouin gain is different as shown in Figure 5(a,b), it is clear that the signal loss increases with increasing pulse width; signal loss in the differential gain case is smaller for larger pulse width difference, while under the Brillouin gain case, the signal loss is higher for smaller pulse width difference. Here the signal loss is defined as:

(33) $$\alpha_{I}=-10\text{log}\frac{I_{\tau 2}-I_{\tau 1}}{I_{\tau 1}}$$

where *I*_*τ*2_ and *I*_*τ*1_ are the Brillouin signal intensities with the pulse widths of *τ*_2_ and *τ*_1_, respectively.

**Figure 6.**
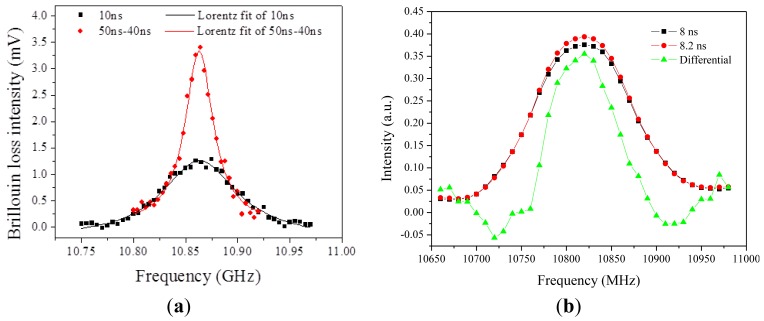
Experimental results of the differential gain for the pulse width larger than phonon lifetime (**a**) and smaller than phone lifetime (**b**).

For the differential gain spectrum of large pulses (>phonon lifetime), the difference spectrum is narrower than the equivalent Brillouin gain spectrum as shown in Figure 6(a). This is also true for the transient Brillouin scattering regime as shown in Figure 6(b). When the pulse width is shorter than the phonon lifetime (10 ns), the Brillouin gain is reduced significantly due to the large pulse spectrum as seen in Figure 5(a), under such a condition, the decrease in differential gain spectral width is smaller than the Brillouin gain spectrum width as seen in Figure 5(b). Hence differential gain provides higher frequency accuracy, i.e. higher temperature or strain resolution compared to that in BOTDA or BOTDR, in which the Brillouin gain spectrum is measured. Because the peak frequency uncertainty is proportional to the bandwidth of the Brillouin spectrum (*Δν_B_*) [75]: $\delta \nu_{B}=\frac{\Delta \nu_{B}}{{\sqrt{2}}^{4}\sqrt{\text{SNR}}}$.

With long sensing lengths of 50 km and 50 cm spatial resolution and 0.7 MHz equivalent Brillouin frequency shift can be achieved, for temperature, it means 0.7 °C and for strain it means 15 μm per meter fiber [92] using coded pulses of RZ format pulse series. The detailed review of long sensing length (>50 km) can be found in reference [2] using different approaches.

Limitations of the long sensing length are gain saturation of the Stokes wave, or equivalently, pump depletion. To avoid this problem, return-to-zero (RZ) coded pulses give better results than NRZ coded pulses due to nonlinear effect induced bit pattern dependence in the signal recovery process, in addition, the continuous (CW) beam must be as low as possible to avoid large depletion induced Brillouin spectrum distortion. As a trade-off of the low Brillouin interaction, lower gain is expected, along with lower signal to noise ratios, as explained in Figure 5 differential Brillouin gain has lower SNR due to the signal subtraction of different pulse widths, and yet it provides higher spatial resolution [92].

The longest sensing length of BOTDA in the report is 150 km with the spatial resolution of 2 m and Brillouin frequency accuracy of 1.5 MHz @1.5 °C/30 με [93]. It was achieved with three different fiber sections of different Brillouin frequencies at normal chromatic dispersion for the purposes of: (1) to avoid intensive Brillouin interaction over the entire sensing length, which will induce the Brillouin spectrum distortion, *i.e.*, depletion effect; (2) to avoid the onset of modulation instability (MI) [94], which is a process by which the amplitude and phase modulation of the wave grow as a result of the interplay between nonlinearity and anomalous dispersion. In the frequency domain MI leads to the generation of sidebands symmetrically placed about the pump frequency, hence the energy in the high power pump or probe waves will be transferred to those sidebands instead of contributing to the Brillouin gain or loss process. The sensing fiber includes two identical spans of fibers being amplified by EDFAs in between them.

### 7.4. Differential of Different Pulses in the Same Waveform

In order to improve spatial resolution, various complicated techniques have to be used in order to enhance the nonlinear Brillouin interaction portion in the same waveform by DC leakage. It was noted by simulation in [95] that the scattering interaction ceases immediately upon the end of the pulse. If the presence of a small CW component in the pulse signal could pre-pump the phonon field before the arrival of a pulse, increased scattering for the duration of the pulse might result, which would make practical use of shorter optical pulses resulting in higher resolution, at the cost of some distortion of the optical signal. Based on this idea, two types of systems have been demonstrated: (1) Dark pulse regime [96]; and (2) Brillouin echoes [97].

When both the pump and probe waves are continuous wave, launched from two ends of the fiber with the frequency difference at the fiber Brillouin frequency, both wave will interact with the pump wave giving energy to the probe wave. If the Stokes wave is suddenly turned off, the pump wave will stop giving energy, and as a result, it will have relatively higher power during times when the Stokes wave is switched off. The period that the Stokes wave is switched off is called “dark pulse”. Based on this idea, 5 cm spatial resolution over 100 m fiber was obtained [96].

The idea of a π-phase-shifted pulse added to a non-phase-shifted pulse in [97] is similar to that of a bright pulse being added to a dark pulse, except that it's based on phase modulation, rather than amplitude modulation. In the π-phase-shifted pulse pair, two pulses share the same pulse-width except that the last portion of the second pulse is phase inverted (π phase shift). As a result, the spatial resolution is limited by the fall-time of the pump modulation and the phenomenon of secondary “echo” signals as was previously proposed in MRI (magnetic resonance imaging).

One of the drawbacks with using large pulse subtraction to get small spatial resolution in the time domain by way of the “echo” or “dark pulse” techniques is the gain saturation of the large pulse portion which decreases the contrast of a small stress or temperature section. Hence the depletion could become a problem for long sensing lengths due to the 1st part of the pulse generating artifacts in the Brillouin spectrum recovery process. To solve this problem a dark base approach is proposed [98], it uses a dark base to remove the strong gain saturation or pulse depletion effect due to the bright pulse. The level of the dark base varies with the width of the bright pulse over the entire sensing length, which makes it difficult to use in the field.

Another technique to overcome depletion induced Brillouin spectrum distortion utilizes coherent interaction of the Brillouin gain and loss [99,100] through a parametric Brillouin gain process, which will be explained in the following section.

The distributed sensor based on Brillouin scattering was initially developed based on spontaneous Brillouin scattering, namely BOTDR, and stimulated Brillouin scattering based BOTDA. The spatial resolution is limited by the acoustic wave delay time (10 ns) [75], which is equivalent to 1 m spatial resolution. Thanks to the innovative approaches of pre-pumping [90,95], differential Brillouin gain [91], and Brillouin echoes [97], distributed Brillouin sensors have achieved comparable spatial resolution to that of OFDR: 2 cm over 2 km and 2 °C temperature resolution [32] while the Rayleigh or stimulated Brillouin scattering based OFDR sensors have limited sensing length of less than 100 m [6,89].

Simultaneous temperature and strain sensing is another challenging issue, because BOTDA and BOTDR measure the change of the Brillouin frequency as a function of temperature and strain based on Equation (32). One variable cannot obviously determine two parameters. Often the intensity or power ratio was introduced [76,79,80] as a 2nd measureable parameter; however the power changes with SOP at different positions, which introduces a new uncertainty by itself, although power ratio can compensate the power fluctuation of light source. As a result, the temperature and strain resolution is often worse than temperature (3–4 °C *vs.* 1 °C) or strain sensing (∼100 με *vs.* 10 με) systems with a single sensing parameter and the spatial resolution is also increased: 10 cm *vs.* 3–5 m. Even if the power measurement is replaced with linewidth dependence in LEAF fiber [101], the temperature resolution has been improved to 1.8 °C and strain resolution of 37 με with 4 m spatial resolution. Unless special fibers are considered, such as in PCF (photonic crystal fiber), two Brillouin peaks represent Ge and Si contributions, and they have different temperature and strain dependence [102]. In PMF as well, the linewidth and peak frequency have different temperature and strain dependence due to the stress rod contribution [103]. In both cases simultaneous measurement can be realized in comparable precision to single parameter sensing in SMF.

## 8. The Parametric Brillouin Gain and Its Application in Birefringence Measurement

When the anti-Stokes wave *ω_AS_*, and Stokes wave *ω_S_*, are launched in one end, and the carrier wave *ω_0_* is launched in the other end of the fiber, there will be two acoustic waves created. These two acoustic waves will interact to form local fringes as their creation is associated with two phase matching conditions of the Brillouin gain and Brillouin loss process [100], and they have different Brillouin frequencies due to the wavelength difference and the different effective indices of each frequency, shown in Equation (15). The carrier wave acts as a receiver and donor for the gain and loss process simultaneously. Such a process can only occur when two phase matching conditions are met at the same time; otherwise, the process will be dominated by either gain or loss. Such a process can be used to offset the gain saturation or pump depletion [99], because the measured Brillouin gain or loss spectrum is similar to the pure gain or loss case.

In PMF if the input SOPs of three waves are launched in the same axis, one can observe the interference spectrum of the parametric gain process [100]. In SMF the coupling between the gain and loss processes change with position; unless the principal state of polarization (PSP) remains the same over certain distances, so that the combined gain and loss spectrum keeps similar a shape with only amplitude change. Parametric Brillouin gain and loss processes require frequency, phase and polarization matching simultaneously, and the amplitude of the signal is only 5–10% of that of the gain or loss dominated process.

The combined gain and loss spectrum has been observed experimentally using SMF-28e, LEAF and dispersion shifted fibers [99,104], where both the Stokes and anti-Stokes pulsed signals were launched in the fiber with the same SOP, created by two identical side bands of an EOM: *ω*_0_ + Δ*ω* = *ω_AS_* [electrical field of *E_AS_* with propagation constant of *k_AS_*]; *ω*_0_ − Δ*ω* = *ω_S_* [electrical field of *E_S_* with propagation constant of *k_S_*], and a CW carrier wave *ω*_0_ [electrical field of *E_0_* with propagation constant of *k_0_*] that is propagated in the opposite direction of the two pulsed wave to meet the phase matching condition for creation of the two excited acoustic waves.

Because Brillouin frequencies are unequal due to their different effective indices at different frequencies (*i.e.*, chromatic dispersion), both of the Brillouin gain and Brillouin loss are not in-resonance simultaneously as stated in Section 2.4, so we name this spectrum the off-resonance Brillouin spectrum. As illustrated in [104] a spatial period of 5 m is observed in LEAF, which represents the process of the gain or loss varying from maximum to minimum. Due to small chromatic dispersion (CD) in LEAF fiber: −3 ps/km/nm [93], the spatial period (the spatial distance for gain changes from maximum to minimum) in gain side is similar to that in loss side (the spatial period for the loss changes from maximum to minimum) as illustrated in experimental results in [104], when the input SOPs for Stokes and anti-Stokes wave are aligned to that of the carrier wave. For SMF28e, both periods can be different and it varies in position due to large CD of 17 ps/km/nm and PMD induced SOP change.

This period is on par with what we have measured with the OFDR technique as described in Section 6. The birefringence calculated from the measured beat length for LEAF is 3 × 10^−7^, which is consistent with the data sheet of the LEAF fiber.

## 9. Brillouin Grating

Brillouin gratings are another example of Brillouin parametric amplification and can be generated in polarization maintaining fiber (PMF) with a length determined by two pump pulses. Such gratings can be generated in one axis and read from another via birefringence coupling with two additional probe waves [105]. It represents a four-wave mixing process at strong pump waves [106]. At relatively weaker pump power, Brillouin gratings have similar features to fiber Bragg gratings (FBGs), *i.e.*, the reflected spectrum is linearly proportional to pump power.

The grating spectrum is a convolution of the pulse and Brillouin gain spectra. Such a Brillouin grating can be probed with a 3rd pulse launched on the orthogonal axis; the optimum frequency of the 3rd pulse is related to the local birefringence between two axes of PMF. This means, the grating feature is affected by temperature and strain reflected in the Brillouin frequency, as well as the local birefringence from two axes of PMF, this local Transient/Dynamic Brillouin Grating (TBG or DBG) [107,108] can be used for the purpose of sensing. Note, TBG refers to the Brillouin grating created by short pulse (<phonon life time). When we change the relative delay between two pump pulses, distributed sensing can be realized for the entire fiber. Because the large frequency shift associated with the birefringence change is on the order of 40–50 GHz, the measurement accuracy of temperature and strain can be much higher than direct Brillouin gain spectrum measurement. It has been reported that 0.08 °C and 3 με resolution can be achieved with a continuous wave (without location information) [107] and pulse of 2 ns, equivalent to 20 cm of spatial resolution, with accuracies of 0.4 °C and 9 με respectively [108]. For BFS (Brillouin frequency shift) measurements, differential pulse-width pair Brillouin optical time-domain analysis (DPP-BOTDA) is used to realize a high spatial resolution. For example, 20 cm spatial resolution is achieved by using a pulse-width difference of 2 ns and a narrowband Brillouin gain spectrum is obtained from a pulse pair of 30/28 ns. For birefringence-induced frequency shift (BireFS) measurements, two short pump pulses (2 ns) are used to generate a local Brillouin grating and thus get a high spatial resolution of 20 cm. The temperature and strain range can be up to 700 °C and 14 mε due to the short pulse (2 ns) based TBG. They can also be used as a distributed birefringence measurement [109] in PMF and PCF [110].

The distributed sensor based on Brillouin grating is often for short sensing length (<500 m) due to the usage of PMF, at long sensing length, the extinction ratio of polarization modes is hard to maintain. Because the Brillouin grating is formed by Brillouin gain or loss process, two lasers must be used to create the TBG, and a 3rd laser is needed as a reading beam with the frequency being locked to the frequency difference of the up or down conversion of the SBS between of the PMF's fast and slow axis. The sensor system is complicated and expensive, and yet it takes long time to scan the Brillouin gain spectrum in one axis with each pulse [108] with the Brillouin grating in another axis. The measurement time can be 10–20 min depending on the length of the grating and probe pulse (recover grating beam).

## 10. Vibration Measurement with Distributed Sensor

Distributed fiber sensors were used mainly for static measurements, such as temperature and strain. Dynamic measurement is more challenging to achieve, as it requires wide frequency range scanning and large scale averaging to improve weak signals. For Rayleigh scattering based OTDR, the first fiber vibration sensor was carried with polarization OTDR achieving 10m spatial resolution and 5 kHz event detection [50]. The best spatial resolution was realized with coherent detection of phase OTDR at 8 kHz and spatial resolution of 0.5 m over 1 km sensing length [56]. With Rayleigh scattering based OFDR sensors, a vibration frequency of 30 Hz has been demonstrated with 10 cm spatial resolution [111]. For Brillouin scattering based sensors, the first demonstration is realized at 8.8 Hz over a 5 cm dynamic strain event based on BOCDA [112]. The field application of impact wave detection was demonstrated on a concrete deck excited by car passing at a frequency of up to 300 Hz based on polarization change on BOTDA [113].

The frequency range of the pulsed OTDR system is the repetition rate of the pulse, which will be limited by the sensing length. For OFDR based CW lasers, it is limited by the speed and memory of the data acquisition card. The signal to noise ratio will limit the achievable spatial resolution.

## 11. Limitations for Sensing Length, Spatial Resolution and Temperature/Strain Resolution

Generally speaking a scattering based OTDR sensor is capable of managing long sensing lengths. Among Rayleigh, Raman and Brillouin scattering based sensors, Brillouin sensors have shown the best performance in terms of sensing length, over 150 km with spatial resolution of 2 m and temperature resolution of 1.5 °C [93] with Er doped fiber amplifier, and high spatial resolution of a few centimeters. The limitations of sensing length result from fiber losses over the long sensing length, which can be compensated by either Raman scattering amplification or EDFAs [2]. The performance of distributed sensors is listed in following Table 2:

Among three different kinds of scattered light in optical fiber, Rayleigh scattering is the strongest, Brillouin scattering is 15–20 dB weaker, and Raman is the weakest. However, when a laser with a 50 kHz linewidth is used as a phase OTDR source, the spectral width of the Rayleigh scattering is the same as that of the laser, while the Brillouin spectrum width is 30 MHz, and the Raman scattering width is ∼THz. One can integrate the optical energy over the spectrum for Brillouin and Raman scattering to improve SNR, while for Rayleigh scattering this is not possible due to the narrow linewidth. Because of Brillouin and Raman gain, the sensing length can be improved significantly.

With high spatial resolution (1 mm) the frequency domain distributed sensor is much more cost effective than OTDR, as the broadband electronics and digitizer requirements have made OTDR based systems very expensive. This is especially true for Brillouin grating type sensors, which requires three lasers to be locked together and PMF to be employed as the sensing fiber which makes such systems complicated and expensive. The advantage of Brillouin scattering based distributed sensors versus Rayleigh scattering based sensor systems are: (1) they measure the Brillouin frequency change, which is an absolute change, rather than the intensity or phase change; and (2) OFDR can only detect relative changes from one of the reference fiber conditions. It uses a correlation function to locate temperature or strain changes between two events. It means that large temperature or strain gradients will not be detected, as the temperature or strain induced wavelength shift can be larger than the wavelength peak itself.

## 12. Challenges in Applications of Distributed Sensors

For a large scale structure, the number of point sensors needed to generate complete strain information can grow rapidly. Distributed sensors offer an advantage over point sensors for global strain measurements. The thousands of sensing points that the distributed sensor provides enables mapping of strain distributions in two or even three dimensions. Thus, real measurements can be used to reveal the global behavior of a structure rather than extrapolation from a few point measurements. Such a process is termed structural health monitoring (SHM), and has been used to identify early signs of potential problems in civil structures, to prevent disasters, and conduct needed repairs at the appropriate time, avoiding unnecessary costs and reducing economic burden. Thus, it is important to have accurate and real time monitoring on the safety assessment of civil structures, such as bridges, dams and pipelines. Currently such evaluations are carried out by engineers trained in visual inspection, which sometimes can be inaccurate due to differences in their personal experience with safety condition assessment. To increase the inspection efficiency and accuracy, fiber optic sensors are one of the most promising candidates, due to their features of durability, stability, small size and insensitivity to external EM perturbations, which makes them ideal for the long-term health assessment of civil structures. Optical fibers can cover the large areas of civil structures, safely access and monitor the status of these structures. Hence, distributed fiber sensor systems will play important roles in the monitoring and diagnostics of critical structures, as they have the advantage of a long sensing range and the capability of providing strain or temperature at every spatial resolution along the entire sensing fiber, imbedded in or attached to the structures, using the fiber itself as the sensing medium. The industrial applications of BOTDR have been reviewed [118], in which the results of three field tests are presented, namely, a damage-detection system for America's Cup yachts, an optical fiber sensor for detecting changes in river levees, and a strain-sensing optical fiber embedded in concrete structures. The extensive review of BOTDA on civil structures of steel pipe and concrete beams has been provided by reference [119]. An excellent example of the prediction of the pipe deformation has been demonstrated [120] in two steel pipes. The major concern with fiber sensors in field applications is the process of installation, as the bare fibers break easily, and cabled fiber has low strain sensitivity, in addition, bending can be introduced in the field installation process, and shear bends especially can be detrimental for the OFDR system due to the weak signal of Rayleigh scattering. The special cable for strain measurement plus the field installation cost can be as high as the sensor system itself; this has been an issue for field applications of distributed sensors.

## 13. Conclusions

This paper presents a comprehensive and systematic overview of the history of distributed sensor technology regarding many aspects, including sensing principles, properties, and their performance, system limitations and applications. It is anticipated that many more distributed sensing systems will be commercialized and widely put into practice in the near future due to the maturity of many new technologies and availability of cost effective instrumentation techniques. The applications of the distributed sensors in civil structural, aerospace and power industry will finally make this technology beneficial to the society after many years of the research work.

## Figures and Tables

**Table 1.** Statistics of PDF of Brillouin frequency with different input SOPs.

**Input SOP** **(Probe)**	**Mean Value** **(MHz)**	**Standard Deviation σ** **(MHz)**	**Skewness** **(Normalized 3rd Order)**	**Kurtosis** **(Normalized 4th Order) (MHz)**	**Mode Value** **(MHz)**	**4σ Bound** **(MHz)**
SOP1	10,861.01	0.045	−0.18	2.88	10,861.03	0.18
SOP2	10,861.00	0.043	0.27	2.70	10,860.98	0.17
Scrambled	10,861.0	0.040	0.043	3.21	10,861.0	0.16

**Table 2.** Performance chart for distributed sensors.

	**DPP-BOTDA** [91]	**Brillouin Grating** [105]	**BOTDR** [68]	**Raman OTDR** [30]	**OFDR** [3] **Rayleigh**	**Phase OTDR** [7] **Rayleigh**
**Spatial resolution**	2 cm (2 km) [32] 2 m (150 km) [93]	1–2 cm [114]	∼1 m	0.4 m [30] 17 m [115]	∼1 mm [10]	∼0.5 m [56]
**Sensing range**	150–200 km	20 m	20–50 km ^a^	900 m [30] 37 km [115]	∼35 m [58]	1–2 km
**Measurement time**	2–5 min	10+ min	1–5 min	<3 min	(0.01–3) s	<1 ms
**Temperature and strain**	Yes	Yes	Yes	No	Yes	No
**Temperature accuracy**	1–2 °C [32,93]	1 °C	2–3 °C	0.8 °C [30] 3 °C [115]	0.1 °C [111]	No
**Strain accuracy**	20 με	10 με	60 με	No	1 με [11]	No
**Dynamic measurement**	yes	No	No	No	30 Hz [111]	Yes 8 kHz [116]
**Calibration**	Determined by fiber property	Determined by fiber property	Determined by fiber property	Relative measurement	Reference is needed for every measurement	Relative measurement
**Light source requirement**	Two DFB lasers [117] (frequency locking of two lasers is required)	Three lasers (frequency locking of two lasers is required)	One narrow linewidth laser	One high power laser	One narrow linewidth tuneable laser	One narrow linewidth laser
**Detectors**	Broadband [32]	Broadband	High sensitivity	High sensitivity	High sensitivity	High sensitivity
**Detection scheme**	Direct	Direct	Coherent	Direct	Coherent	Coherent [56] Direct [116]

a Yokogawa Electric Corporation.
